# Analysis of the Effect of External Counterpulsation Combined With High-Intensity Aerobic Exercise on Cardiopulmonary Function and Adverse Cardiovascular Events in Patients With Coronary Heart Disease After PCI

**DOI:** 10.3389/fsurg.2022.851113

**Published:** 2022-03-03

**Authors:** Shiming Zhao, Shaowen Liu, Yuan Wen, Qiuhuan Qi, Peng Huang

**Affiliations:** ^1^Department of Cardiology, Wuhan Hankou Hospital, Wuhan, China; ^2^Intensive Care Unit, Emergency Medical Department, Wuhan Hankou Hospital, WuHan, China

**Keywords:** coronary heart disease, external counterpulsation, high-intensity aerobic exercise, cardiopulmonary function, adverse cardiovascular events

## Abstract

**Purpose:**

To explore the intervention effect of external counterpulsation (ECP) combined with high-intensity aerobic exercise (HIAT) on patients with coronary heart disease (CHD) after PCI.

**Methods:**

124 patients with stable CHD after PCI admitted to our hospital from June 2018 to June 2021 were selected, and all patients were divided into control group and observation group using the random number table method. The control group received conventional treatment, The observation group received ECP combined with HIAT based on the control group. The cardiorespiratory function indexes, exercise endurance indexes, incidence of major cardiovascular adverse events (MACE), Barthel index of the two groups were observed.

**Results:**

After intervention, METs _max_, VO_2__max_, VO_2__max_/kg, VO_2__max_/HR, and PP, ED, AT, and Barthel score in both groups were significantly higher than before intervention, and patients in the observation group were significantly higher than those in the control group (*P* < 0.05). The incidence of MACE in the observation group (3.23%) was lower than in the control group (12.90%) (*P* < 0.05).

**Conclusion:**

ECP combined with HIAT can improve the cardiopulmonary function of patients with CHD after PCI, and improve exercise endurance, reduce the incidence of MACE, improve patients' ability of daily living.

## Introduction

Coronary heart disease (CHD), a common disease among middle-aged and elderly people, has become the leading cause of hospitalization and death in China. The onset age of this disease is generally after 60 years old, and in recent years, the prevalence of CHD has been on a rapid rise (1). With the development of science and technology and medical treatment, percutaneous coronary intervention (PCI) is increasingly used in the treatment of CHD, which is a therapeutic method for patients with coronary artery stenosis to unblock the narrowed or occluded coronary artery lumen by transcatheter technique. It has the advantages of less trauma, quick recovery and high success rate (2, 3). However, PCI is not the end of treatment for patients with CHD. Although PCI can save patients' lives, the incidence of major cardiovascular adverse events (MACE) after PCI is high and the recovery of cardiopulmonary function after PCI is poor (4, 5). At present, only drug or surgical treatment can not completely relieve the risk factors of patients with CHD, and it is of great clinical significance to effectively stabilize the condition of patients with CHD, reduce the incidence of coronary complications, and improve the cardiopulmonary function of patients.

Research has shown that the key to improving the quality of life and prognosis of patients with CHD is not only conventional drug therapy, but also somato-psychological and other integrated rehabilitation measures are equally important (6). External counterpulsation (ECP) is a non-invasive assisted circulation device, which sequentially inflates the balloon during the diastolic phase of the heart to promote blood return to the lower extremity arteries and increase coronary artery perfusion, and is beneficial to improving myocardial blood supply and increasing oxygen-carrying capacity, thus affecting cardiopulmonary function, and has become the main non-drug treatment for various angina pectoris, heart failure and other cardiovascular diseases (7, 8). In addition, cardiac rehabilitation therapy with exercise training as the core content is gradually recognized and respected by clinical health care professionals and patients. High-intensity aerobic training (HIAT) can reduce the body's inflammatory reaction, improve the patient's endothelial function, promote the establishment of coronary collateral circulation and delay coronary stenosis through high-intensity effective exercise stimulation (9). HIAT not only helps to control body weight, improve patients' blood pressure and blood glucose, but also prevents cardiovascular events, promotes mental health, and controls risk factors of cardiovascular disease as a whole, thus improves patients' exercise function and survival quality, and has a positive impact on patients' prognosis (10). The aim of this study was to investigate the effect of ECP combined with HIAT on cardiopulmonary function and MACE in patients with CHD after PCI.

## Materials and Methods

### Object

124 patients with stable CHD after PCI admitted to our hospital from June 2018 to June 2021 were selected, and all patients were divided into control group and observation group using the random number table method, with 62 cases each.

### Inclusion Criteria

Met the diagnostic criteria of coronary heart disease (11); PCI was performed successfully for the first time within 3 months; Hemodynamics was stable after PCI; Have the condition of basic movement.

### Exclusion Criteria

Accompanied by movement restriction diseases such as bone joints and muscles; Patients with severe arrhythmia and severe heart failure that affect ECP; Severe cardiopulmonary dysfunction; Those who were unable to perform cardiopulmonary exercise test for various reasons; Accompanied by systemic serious organic diseases; Complicated infectious diseases; Mental disorder, abnormal cognitive function, Unable to cooperate with training; Increase or decrease the amount of exercise if you did not follow the instructions.

### Methods

The control group received conventional treatment, including drug therapy, anti-blocking rehabilitation training, and daily nursing (1). The medical staff gave the patients anti-platelet aggregation, nitrates, angiotensin-converting enzyme inhibitors, and statins (2). Integrated with the guidance of the director of our rehabilitation department, the patients performed elastic band exercises with the help of researchers to ensure that the patients did not feel any discomfort on the day of training, and instructed the patient to wear a heart rate monitor. Preparatory activities and relaxation activities were performed before exercise, relaxation movements and warm-up movements include shoulder, wrist, ankle, neck, waist, hip, knee joint activities. The patients' blood pressure and heart rate were closely monitored during exercise, and exercise was stopped immediately if symptoms such as progressive chest pain, pale complexion, ataxia, dizziness, fatigue, and shortness of breath occurred. The training forms could simply be arranged and designed according to the movement of the joint, the resistance provided by the elastic band at 100% extension was 1.7 kg. In resistance training, each isometric contraction lasted 10s, rested for 10 s, repeated 10 times as a set of training, and each training was done with 10 sets of training (3). Health education was carried out on quitting smoking and drinking, eating regularly, exercising properly, and regulating emotions.

The observation group received ECP combined with HIAT based on the control group. Patients were evaluated by cardiopulmonary exercise test before the intervention. Patients were first warmed up with a power bike for 5 min with no load and rested for 3 min with an initial power of 5 W. The power was increased at a rate of 10 W/min. Patients were kept at a speed of 50–60 r/min while pedaling training. When patients had chest pain, weakness, dyspnea and other uncomfortable symptoms, or when ECG and blood pressure monitoring reached the indications for test discontinuation, the evaluation was discontinued and peak power (PP) was recorded (1). ECP: The intervention was performed with a balloon type ECP device (P-ECP/TM, Pushkang, Chongqing). During the treatment, the patient was lying flat on the bed, and airbags were pumped on the patient's calves and thighs as well as buttocks, which were connected to the air compressor through an air tube. Under cardiac monitoring, the balloons were inflated and deflated simultaneously with the patient's cardiac cycle, with sequential compression of the lower limbs and buttocks during diastole and rapid deflation of the three balloons during systole, with a counterpulsation balloon inflation pressure of 260–340 mmHg and a finger pulse wave showing a diastolic/systolic wave ratio >1.2. 1 time/d, 1 month was a course of treatment (2). HIAT: After 5 min of warm-up, patients were trained with power treadmill by bicycle with aerobic exercise intensity of 80% PP, 3 min for each group, with 1 min rest between groups, 10 groups for each training, a total of 40 min. The initial training could be carried out with 60% PP as exercise load for 7 days of adaptive training. The treatment lasted for 3 months, 1 time/d, and 3 times/week.

### Observation Index

(1) Baseline information such as patient's age, gender, smoking history, alcohol history, combined diseases, and postoperative course of PCI were recorded.
(2) Before intervention and 3 months after intervention, the K482 cardiopulmonary exercise test training system (COSME, Italy) was used to measure the patients' cardiorespiratory function indexes. The patients' maximal METs (METs _max_), maximal oxygen uptake (VO_2__max_), maximal oxygen uptake every kilogram (VO_2__max_/kg) and maximal oxygen pulse (VO_2__max_/HR) were recorded.
(3) Before intervention and 3 months after intervention, the K482 cardiopulmonary exercise test training system (COSME, Italy) was used to measure the exercise endurance indexes of the patients. The PP, exercise duration (ED) and anaerobic threshold (AT) in the patients' cardiopulmonary exercise test were recorded.
(4) The incidence of MACE such as angina pectoris, arrhythmia and heart failure was recorded in both groups within 3 months of intervention.
(5) Before intervention and 3 months after intervention, the Barthel index was used to evaluate the patients' ability of daily living. The scale had 10 items with a total score of 100 points, >60 points: in daily life, patients could basically take care of themselves; 40–60 points: in daily life, patients needed the help from others; 20–40 points: life needs a lot of help; <20 points: in daily life, patients completely needed the help from others. The higher the score, the stronger the independence and the smaller the dependence of the patient.

### Statistical Methods

SPSS 22.0 software was used for analysis. The measurement data was (± s), the comparison was made by *t*-test, the count data was (%), and the comparison was made by χ^2^ test. *P* < 0.05 was statistically significant.

## Results

### Baseline Information of the Patient

There was no statistical difference in age, gender, smoking history, alcohol history, combined diseases, and postoperative course of PCI between the two groups (*P* > 0.05). As shown in Table 1.

**Table 1:** Baseline information of patients (*n*, %, $\bar{x}\pm$ s).

**Group**	**Number of cases**	**Age (years)**		**Gender**		**Smoking history**	**Alcohol history**
		**<60**	**≥60**	**Male**	**Female**		
Control group	62	28 (45.16%)	34 (54.84%)	31 (50.00%)	31 (50.00%)	36 (58.06%)	35 (56.45%)
Observation group	62	30 (48.39%)	32 (51.61%)	27 (43.55%)	35 (56.45%)	37 (59.68%)	39 (62.90%)
*χ^2^* value		0.130		0.518		0.033	0.536
*P-*value		0.719		0.472		0.855	i0.464
**Group**	**Number of cases**	**Combined diseases**				**Postoperative course of PCI (d)**
		**Diabetes**	**Hypertension**	**Hyperlipidemia**	
Control group	62	19 (30.64%)	14 (22.58%)	13 (20.96%)		40.23 ± 8.13
Observation group	62	17 (27.42%)	16 (25.81%)	12 (19.35%)		38.85 ± 8.55
*χ^2^/t* value		0.273				0.920
*P-*value		0.872				0.359

### Cardiopulmonary Function of Patients

**Figure 1:**
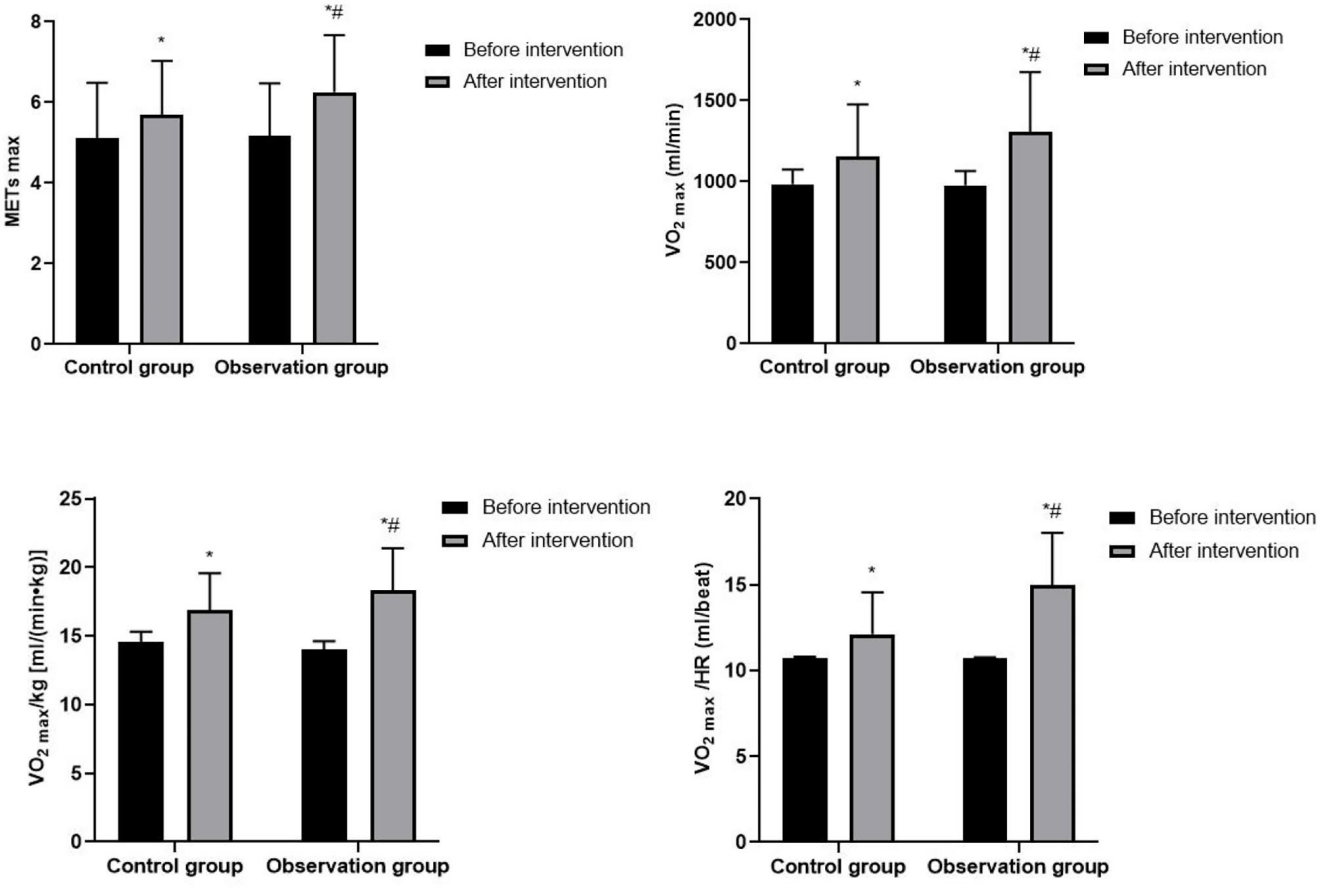
Cardiopulmonary function of patients. Compared with before intervention, *P < 0.05; compared with control group, ^#^*P* < 0.05.

After intervention, METs _max_, VO_2__max_, VO_2__max_/kg, and VO_2__max_/HR in both groups were significantly higher than before intervention, and patients in the observation group were significantly higher than those in the control group (*P* < 0.05). As shown in Figure 1.

### Exercise Endurance of Patients

**Figure 2:**
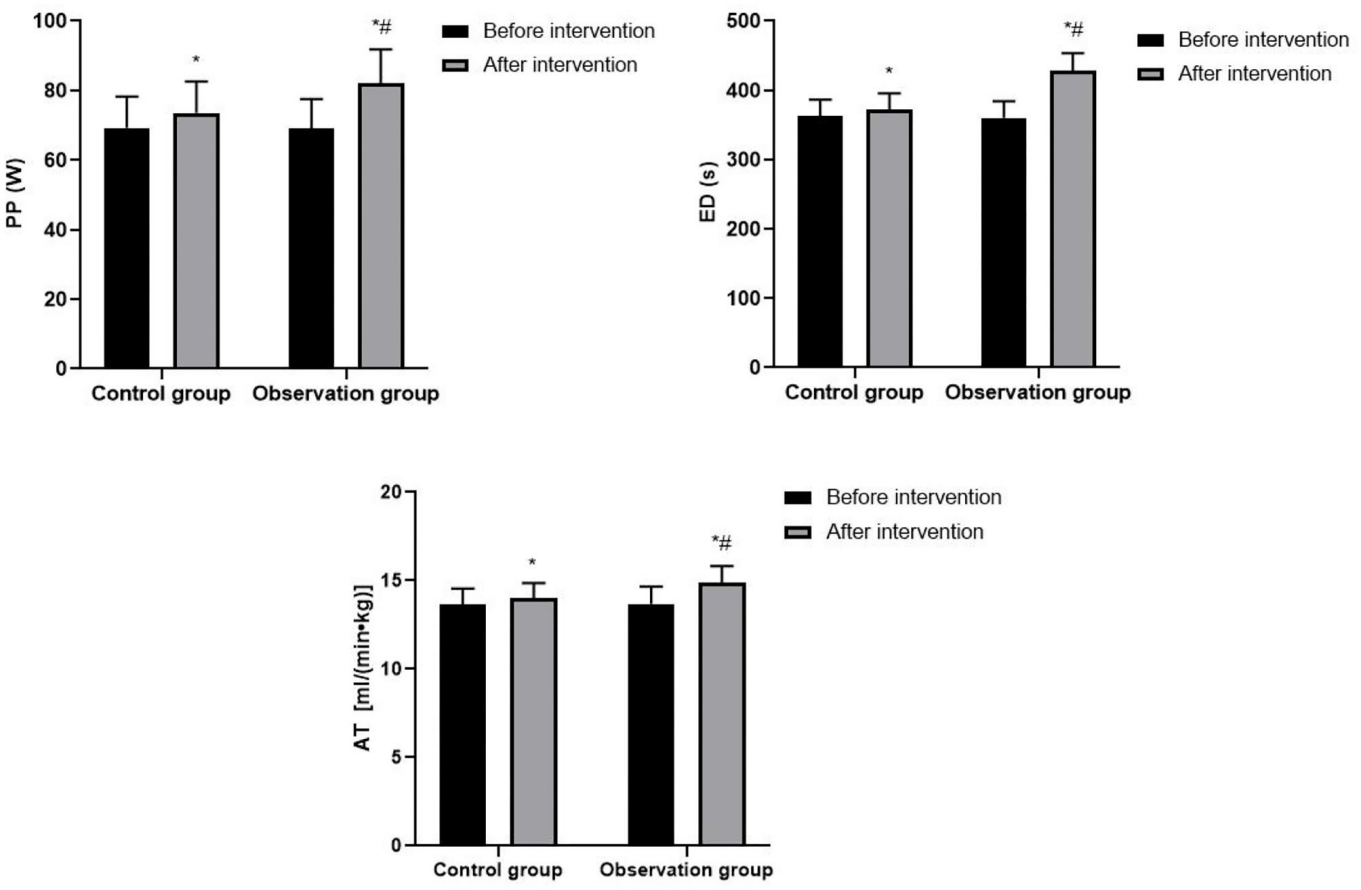
Exercise endurance of patients. Compared with before intervention, *P < 0.05; compared with control group, ^#^*P* < 0.05.

After intervention, PP, ED, and AT in both groups were significantly higher than before intervention, and patients in the observation group were significantly higher than those in the control group (*P* < 0.05). As shown in Figure 2.

### Incidence of MACE in Patients

The incidence of MACE in the observation group (3.23%) was lower than in the control group (12.90%) (*P* < 0.05). As shown in Table 2.

**Table 2:** Incidence of MACE in patients (*n*, %).

**Group**	**Number of cases**	**Angina pectoris**	**Arrhythmia**	**Heart Failure**	**Total incidence**
Control group	62	5 (8.06%)	2 (3.23%)	1 (1.61%)	8 (12.90%)
Observation group	62	1 (1.61%)	1 (1.61%)	0 (0.00%)	2 (3.23%)
*χ^2^* value					3.916
*P-*value					0.048

### Ability of Daily Living of Patients

**Figure 3:**
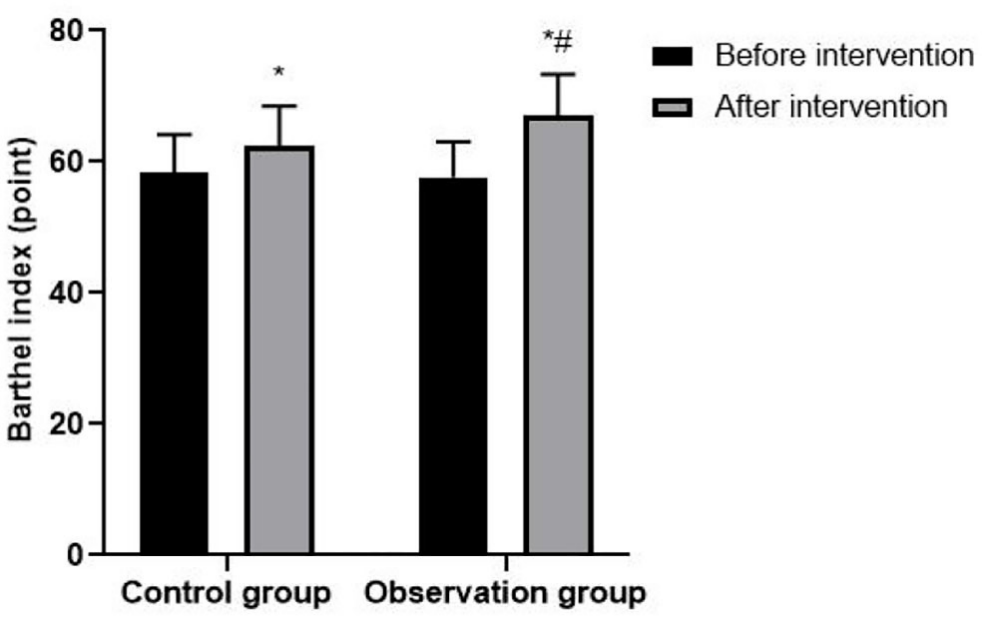
Ability of daily living of patients. Compared with before intervention, *P < 0.05; compared with control group, ^#^*P* < 0.05.

After intervention, the Barthel score in both groups were significantly higher than before intervention, and patients in the observation group was significantly higher than that in the control group (*P* < 0.05). As shown in Figure 3.

## Discussion

PCI is one of the common clinical treatment modalities for CHD, which can effectively improve myocardial blood perfusion, promote myocardial cell recovery and improve prognosis (12). However, after PCI, the myocardial blood supply of patients with CHD is insufficient, and the oxygen-carrying capacity of the body is reduced, which leads to the decline of cardiopulmonary function and exercise endurance, easily triggers MACEs such as angina pectoris, arrhythmia, heart failure, seriously affecting the physical and mental health and life safety of patients (13). At present, the clinic attaches great importance to the rehabilitation of patients with CHD, and the intervention model with the ultimate goal of improving cardiopulmonary function, improving quality of life and returning to society is gradually applied widely.

ECP is a non-medical, non-invasive physiotherapy method that increases cardiac perfusion by wrapping the patient's buttocks and lower extremities with segmental balloons. During the diastolic phase of the heart, the balloons are sequentially inflated to promote the return of blood from the arteries of the lower extremities to the aorta and then to the arteries at all levels, thereby increasing diastolic pressure, and during the systolic phase of the heart, the balloons are rapidly deflated to allow rapid flow of blood from the aorta to the lower extremities to reduce cardiac afterload (14). The principles of ECP therapy are mainly: (1) Increase aortic diastolic pressure, increase coronary blood perfusion and improve myocardial blood supply. (2) Reduce peripheral resistance, improve blood flow, and promote the formation of coronary collateral circulation. (3) Increase the shear stress of blood flow, improve the shape and function of vascular endothelial cell, repair damaged vascular endothelium, and inhibit the development of atherosclerosis. (4) Accelerate blood flow, reduce blood viscosity, improve microcirculation while increasing the oxygen uptake capacity of the body. (5) When the balloon is constantly squeezing the lower limbs, the body's nervous system generates micro-electrical stimulation, which is conducive to relieving muscle tension and relaxing the cerebral cortex (15–17). ECP is a non-invasive, safe, effective, and inexpensive treatment device that can reduce the discomfort of patients with CHD, control the progression of the disease, and change the exercise endurance of the patient, thereby facilitating adaptation to more intense or longer exercise (18). Physical inactivity is one of the risk factors for CHD, and long-term physical inactivity may lead to a decrease in cardiorespiratory fitness, which in turn may affect the patient's quality of life. HIAT can positively affect the cardiovascular system of patients with CHD after PCI in many ways: (1) HIAT can promote the formation of cardiac collateral circulation, improve coronary artery blood supply and intrinsic myocardial contractility, increase coronary blood flow and capillary diffusion, and improve the circulation transportation capacity of the coronary artery, thereby reducing cardiac work and improving left ventricular myocardial function. (2) HIAT can promote adaptive changes in the structure, function and regulatory capacity of the cardiovascular system and skeletal muscle system, which can increase the density of skeletal muscle capillaries, increase the number of myocardial capillaries, improve the supply of peripheral blood, increase the oxygen uptake capacity of skeletal muscle, so as to meet the body's demand for oxygen and reducing the load on the heart. (3) Aerobic exercise can increase the shear stress of coronary blood flow, stimulate the production and release of nitric oxide synthase in vascular endothelial cells, improve the vasodilatory capacity of endothelial intact coronary arteries, improve the function of peripheral vascular endothelial cells, and thus increase myocardial perfusion. (4) HIAT enhances the oxygen utilization capacity and aerobic metabolism of muscle groups, improves mitochondrial function of cardiomyocytes, which in turn increases cardiovascular effects, improves overall patient function, and reduces the incidence of cardiovascular events. (5) HIAT reduces coronary stent lumen loss in patients with CHD after PCI, and this may be closely related to a reduction in the patient's systemic inflammatory response (19–21). Villelabeitia-Jaureguizar have found that compared with moderate-intensity aerobic exercise, although the patients are more laborious during HIAT, the duration of HIAT is short, and interval rest can avoid excessive fatigue and discomfort, which makes the patient's tolerance higher (22). At the same time, HIAT brings stronger exercise stimulation to patients, and the higher the intensity of exercise, the higher the cardiorespiratory fitness of patients with CHD. METs _max_ can reflect the level of cardiac energy metabolism and exercise capacity; VO_2__max_ indicates the body's maximum aerobic metabolic capacity, cardiac output and cardiac reserve function, and VO_2__max_ is the gold standard for evaluating cardiopulmonary function; VO_2__max_/kg corrects the effect of body weight on oxygen uptake and was a predictor of cardiovascular events; VO_2__max_/HR can reflect the oxygen intake capacity of the heart's stroke volume. PP is the maximum exercise load that the patient can tolerate in the cardiopulmonary exercise test; ED is the exercise time that the patient lasted from the beginning to the end of the cardiopulmonary exercise test evaluation; AT is the critical value of the transition from aerobic metabolism to anaerobic metabolism when the body performs increasing load exercise, which can reflect the body's maximum aerobic exercise capacity. In this study, METs _max_, VO_2__max_, VO_2__max_/kg, VO_2__max_/HR, PP, ED, and AT of patients in the observation group were significantly higher than those in the control group, suggesting that ECP combined with HIAT can improve the cardiopulmonary function and exercise endurance of patients with CHD after PCI.

In addition, we found that patients with CHD after PCI had a lower incidence of MACE and better daily living ability after interventions. The traditional single rehabilitation training model cannot provide sufficient training volume to resist the patient's physical strength loss and cannot achieve the goal of motor learning optimization through sufficient repetitive activities, so the therapeutic effect is limited. In contrast, ECP and HIAT can improve myocardial oxygen supply, enhance the physical performance of patients, and relieve or even reduce the occurrence of angina pectoris and arrhythmias. The combined application of the two methods will eliminate obesity and bad mood and other risk factors of cardiovascular and cerebrovascular diseases, help patients gradually recover their ability to perform activities of daily living and improve the quality of survival (23, 24). It is worth mentioning that patients with contraindications to exercise can also be treated with ECP. Clinicians can give ECP to patients with CHD first, and then start HIAT when the patient's condition is stable and there is no discomfort, which is safe and effective in the field of CHD rehabilitation.

## Conclusion

In conclusion, ECP combined with HIAT can improve the cardiopulmonary function of patients with CHD after PCI, and improve exercise endurance, reduce the incidence of MACE, improve patients' ability of daily living. This intervention brings a new model for cardiac rehabilitation. In this study, in order to ensure the uniformity of aerobic exercise intervention intensity for patients, we only used one form of exercise to train patients. In addition, when performing cardiopulmonary exercise test, due to insufficient exercise cooperation and subjective exercise effort of patients, this may affect the research results. At the same time, cardiopulmonary exercise test also require relatively high operation requirements for professional technicians. Therefore, this study needs to expand the sample size, prolong the observation time, and choose the exercise form according to the patient's personal interests in the future, so as to further prove the long-term efficacy of ECP combined with HIAT.

## Data Availability Statement

The original contributions presented in the study are included in the article/supplementary material, further inquiries can be directed to the corresponding author/s.

## Ethics Statement

The studies involving human participants were reviewed and approved by the Ethics Committee of the WuHan HanKou Hospital. The patients/participants provided their written informed consent to participate in this study.

## Author Contributions

PH was the director of the entire study. All authors of this study made equal contributions, mainly including the design of the study, the inclusion of cases, the detection of results, the statistics of the data, and the writing of the paper.

## Conflict of Interest

The authors declare that the research was conducted in the absence of any commercial or financial relationships that could be construed as a potential conflict of interest.

## Publisher's Note

All claims expressed in this article are solely those of the authors and do not necessarily represent those of their affiliated organizations, or those of the publisher, the editors and the reviewers. Any product that may be evaluated in this article, or claim that may be made by its manufacturer, is not guaranteed or endorsed by the publisher.
